# Predictive Coding or Just Feature Discovery? An Alternative Account of Why Language Models Fit Brain Data

**DOI:** 10.1162/nol_a_00087

**Published:** 2024-04-01

**Authors:** Richard Antonello, Alexander Huth

**Affiliations:** Department of Computer Science, University of Texas at Austin, Austin, TX, USA

**Keywords:** encoding models, predictive coding, language models

## Abstract

Many recent studies have shown that representations drawn from neural network language models are extremely effective at predicting brain responses to natural language. But why do these models work so well? One proposed explanation is that language models and brains are similar because they have the same objective: to predict upcoming words before they are perceived. This explanation is attractive because it lends support to the popular theory of predictive coding. We provide several analyses that cast doubt on this claim. First, we show that the ability to predict future words does not uniquely (or even best) explain why some representations are a better match to the brain than others. Second, we show that within a language model, representations that are best at predicting future words are strictly worse brain models than other representations. Finally, we argue in favor of an alternative explanation for the success of language models in neuroscience: These models are effective at predicting brain responses because they generally capture a wide variety of linguistic phenomena.

## INTRODUCTION

Predictive coding is a cognitive theory of the high-level mechanisms underlying sensory processing in the brain. It holds that the brain is constantly attempting to predict future events before they occur. These predictions are revised and updated via error signals generated upon comparison of predictions with observations. Predictive coding is attractive as a theory because it provides a concrete, conceptually simple, and mechanistically plausible objective for brain processing that seems to also relate to our own introspective experience of what it feels like to learn. Although originally formulated to explain visual processing in the brain (Huang & Rao, 2011; Jiang & Rao, 2021; Rao & Ballard, 1999), this theory has also been extended to language processing. For language, predictive coding theories posit that the brain works to preemptively generate predictions about future words and sentences as it perceives natural language stimuli.

Evidence for predictive coding in language processing comes from several strands of research. First, many studies have shown electrophysiological signals associated with syntactically or semantically incongruent words or surprisal (Frank et al., 2015; Gagnepain et al., 2012; Heilbron et al., 2022; Kuperberg & Jaeger, 2016; Kutas & Hillyard, 1984; Münte et al., 1990; Schmitt et al., 2021; Shain et al., 2020). These signals are thought to correspond to “prediction error” between what was predicted and what actually occurred.

Second, many recent studies have shown that neural network language models (NNLMs), which embody (some elements of) predictive coding theory, are much more effective at explaining brain activity elicited by natural language than earlier methods (Anderson et al., 2021; Antonello et al., 2021; Caucheteux et al., 2021a; Caucheteux & King, 2022; Goldstein et al., 2021; Jain & Huth, 2018; Jat et al., 2019; LeBel, Jain, & Huth, 2021; Li et al., 2021; Schrimpf et al., 2021; Tikochinski et al., 2021; Toneva et al., 2020). Some of these studies claim that the superiority of NNLMs over other methods is evidence for predictive coding theory in language (Goldstein et al., 2021; Schrimpf et al., 2021). We argue in this paper that the high performance of these models should not be construed as positive evidence in support of a theory of predictive coding. As an alternative, we propose that the prediction task which these NNLMs attempt to solve is simply one way out of many to discover useful linguistic features.

### Language Models and Encoding Models

Unidirectional NNLMs are artificial neural networks that are trained to perform a “next word prediction” task (Dai et al., 2019; Radford et al., 2019). Specifically, these neural networks are trained to generate a probability distribution over the next word in a sequence, conditioned on a context consisting of previous words. For example, when fed the context “Better late than”, a language model might assign a high probability to the next word being “never.”

Compared to tasks that require labeled data, such as translation, question answering, or word sense disambiguation, NNLMs have a distinct advantage because of the near-limitless amount of data that can be used to train them; almost any natural language text that can be scraped from the internet is valid data to train an NNLM. Further, in order to do effective next-word prediction, NNLMs need to capture a great deal about the statistical regularities in natural language, including everything from part of speech (Tsai et al., 2019) to topic (Sun et al., 2019) to coreference information (Joshi et al., 2019). The ease of training NNLMs and their ability to learn many types of statistical dependencies has, in recent years, developed into the paradigm of *language model fine-tuning*. In this paradigm, representations extracted from existing NNLMs are retooled for other linguistic tasks such as named entity recognition (Li et al., 2020), summarization (Nikolich et al., 2021), question answering (Su et al., 2019), and sentiment analysis (Socher et al., 2013). Fine-tuning from NNLMs often outperforms models that are trained from scratch on these tasks, as it allows the model to reuse linguistic features that were learned by the original NNLM, and helps make up for the limited and costly hand-labeled training data that many downstream tasks currently require (Dodge et al., 2020).

State-of-the-art NNLMs are typically organized into a series of architecturally homogeneous layers of processing blocks called *transformers* (Radford et al., 2019; Vaswani et al., 2017). Transformers use a mechanism known as *dot product attention* to selectively process some elements of their input context while ignoring others. This mechanism enables models to integrate information over much longer timescales than other methods (Vaswani et al., 2017). The output of each transformer layer is an encoded representation of its inputs, often called a *hidden state*. For example, in the commonly used GPT-2 Small model (Radford et al., 2019), the hidden state is a 768-dimensional vector. This output vector is then fed into the next layer as its input. These layers serve to transform information from the initial input (often provided as word embeddings; see Mikolov et al., 2013) to a next word prediction output at the last layer. For this reason, the hidden states of later layers (those near the output) generally tend to act as representations that are more suitable for next word prediction than the hidden states of earlier layers, which are more similar to the initial word embeddings. Language models are typically evaluated by a metric known as *perplexity*, which measures how well they can predict next words. Low perplexity means that the model assigns a high probability to the actual next word, while high perplexity means that it assigns a low probability; that is, lower perplexity is better.

Drawing on the success of NNLMs for transferring to many different language tasks, neuroscientists have used NNLM representations that encode linguistic context to predict brain responses to natural language (Jain & Huth, 2018). Regression models that attempt to predict brain response to natural language stimuli by using an analytic feature space derived from the stimuli can be called *encoding models* (Huth et al., 2016; Naselaris et al., 2011). Much recent work has examined the extent to which features generated by language models can be used as encoding model inputs (Caucheteux & King, 2022; Schrimpf et al., 2021). Particular interest has been afforded to these LM-based encoding models, as they appear to outperform previous approaches that used representations sourced from non-contextual word embedding spaces.

The success of this approach raises a key question: Why do LM-based encoding models perform so much better than encoding models that use other feature spaces? One hypothesis is that these features work so well precisely because their training objective—next word prediction—is the same objective that the brain has learned to solve. For example, both Schrimpf et al. (2021) and Caucheteux and King (2022) showed that there is a strong correlation between encoding model performance for a feature space and that feature space’s capacity for next word prediction. Schrimpf et al. (2021) in particular argue that this strong correlation may be taken as evidence that the next-word prediction task is a fundamental part of biological language processing. Accepting this argument requires us to interpret correlation as causation: Some representations have high encoding performance because they have high next-word prediction performance.

Goldstein et al. (2021) went even further, showing that embeddings for future words can be predicted at significantly above chance by brain responses before word onset, even if simple contextual and semantic information such as word meaning and bigram information is removed. Caucheteux et al. (2021b) demonstrate a similar result, showing that embeddings of future words improve LM-based encoding models over using only present context. They each suggest that these results stand as strong direct evidence of predictive coding in the brain during language processing.

In this article, we analyze the strength of the evidence that encoding model research provides for the theory of predictive coding. We claim that existing evidence does not favor predictive coding above alternative explanations. (However, we distinguish this evidence from the theory of predictive coding itself: It is plausible that the brain is doing predictive coding even if it cannot be proven using this type of evidence.) Our claim is based on two major arguments.

First, we examine the correlation between next word prediction performance and encoding performance and present an alternative hypothesis for why representations from NNLMs perform well as encoding model inputs. In this alternative hypothesis, we suggest that the high encoding performance of NNLM representations can be explained by the fact that these representations transfer effectively to representations from many other linguistic tasks, a quality which is acknowledged in the fine-tuning literature. We produce a standardized metric for this “general” transfer performance and show that it is well correlated with brain encoding performance. We construct another metric that captures transfer performance to a representation extracted from a machine translation model from English to German. We show that the correlation between this translation metric and next word prediction performance is also high, and use this to argue that one should be generally skeptical of drawing strong inferences from correlations with encoding performance alone.

Second, we argue that a theory of predictive coding implies that language representations that are more useful for next word prediction should in general be better at predicting brain responses when controlling for other factors. Caucheteux and King (2022) analyzed the performance of individual layer hidden states as encoding model input features and showed that the intermediate layers of these language models, which are not the best at next word prediction, consistently outperform early and later layers as encoding model features. Using a variance partitioning argument, we build on this result to show that the late representations from NNLMs, which are the best at predicting next words, explain strictly less variance in nearly every cortical voxel than intermediate representations that are less effective at predicting next words. Using these results, we further argue that the existence of predictive information in the brain does not inherently necessitate a theory of predictive coding.

## MATERIALS AND METHODS

### MRI Data Collection

We used functional magnetic resonance imaging (fMRI) data collected from five human subjects as they listened to English language podcast stories over Sensimetrics S14 (2022) headphones. Subjects were not asked to make any responses, but simply to listen attentively to the stories. For encoding model training, each subject listened to approximately 5 hr of unique stories across five scanning sessions, yielding a total of 9,189 data points for each voxel across the whole brain. For model testing, the subjects listened to the same test story once in each session (i.e., five times). These responses were then averaged across repetitions. Functional signal-to-noise ratios in each voxel were computed using the mean-explainable variance method from Nishimoto et al. (2017) on the repeated test data. Only voxels within 8 mm of the mid-cortical surface were analyzed, yielding roughly 90,000 voxels per subject. Language-responsive voxels were identified as those where at least 5% of the response variance for the test story, which was played at least five times for each subject, could be explained by the average response across repetitions (Nishimoto et al., 2017).

MRI data were collected on a 3T Siemens Skyra scanner at the University of Texas at Austin Biomedical Imaging Center using a 64-channel Siemens volume coil. Functional scans were collected using a gradient echo-planar imaging sequence with repetition time (TR) = 2.00 s, echo time (TE) = 30.8 ms, flip angle = 71°, multiband factor (simultaneous multislice) = 2, voxel size = 2.6 mm × 2.6 mm × 2.6 mm (slice thickness = 2.6 mm), matrix size = 84 × 84, and field of view = 220 mm. Anatomical data were collected using a T1-weighted multi-echo MP-RAGE sequence with voxel size = 1 mm × 1 mm × 1 mm following the Freesurfer morphometry protocol (Fischl, 2012).

All subjects were healthy and had normal hearing. The experimental protocol was approved by the Institutional Review Board at the University of Texas at Austin. Written informed consent was obtained from all subjects.

### fMRI Preprocessing

All functional data were motion corrected using the FMRIB Linear Image Registration Tool (FLIRT) from FSL 5.0 (Jenkinson & Smith, 2001). FLIRT was used to align all data to a template that was made from the average across the first functional run in the first story session for each subject. These automatic alignments were manually checked for accuracy.

Low frequency voxel response drift was identified using a second order Savitzky-Golay filter (Savitzky & Golay, 1964) with a 120 s window and then subtracted from the signal. To avoid onset artifacts and poor detrending performance near each end of the scan, responses were trimmed by removing 20 s (10 volumes) at the beginning and end of each scan, which removed the 10 s silent period and the first and last 10 s of each story. The mean response for each voxel was subtracted and the remaining response was scaled to have unit variance.

### Encoding Model Construction

We used the fMRI data to generate voxelwise brain encoding models for 97 different language representations. In order to temporally align word times with TR times, we applied Lanczos interpolation together with a finite impulse response model as described in Huth et al. (2016). Let *t*_*i*_ (𝒮) correspond to the instantiation of the *i*^th^ representation on our transcribed stimulus set 𝒮. Let *g*(*t*_*i*_(𝒮)) indicate a linearized ridge regression model that uses a temporally transformed version of the representation instantiation *t*_*i*_(𝒮) as predictors. The temporal transformation accounts for the lag in the hemodynamic response function (Huth et al., 2016; Nishimoto et al., 2011). We use time delays of 2, 4, 6, and 8 s of the representation to generate this temporal transformation. For each subject *x*, voxel *v*, and representation *t*_*i*_, we fit a separate encoding model *g*_(*x*,*v*,*t*_*i*_)_ to predict the BOLD response $\hat{B}$ from our represented stimulus, that is, $\hat{B}$_(*x*,*v*,*t*_*i*_)_ = *g*_(*x*,*v*,*t*_*i*_)_*t*_*i*_(𝒮). Encoding model performance for a representation was computed as the average voxelwise performance across our five subjects.

### Next-Word Prediction Performance

We performed a linear regression between each representation and the GloVe embedding of the next word (Pennington et al., 2014). We then computed the exponentiated average cross entropy between the distribution over the predicted next word from this regression against the ground truth next word. This value is used as a metric for how well each representation predicts next words. This metric was computed using a test corpus of approximately 54,000 words consisting of transcribed podcasts (LeBel, Wagner, et al., 2021).

### Representational Generality

For our 97 representations, we used the method and publicly available data and code from our earlier work (Antonello et al., 2021) to measure the overall generality of the information contained in these representations. Let 𝒮 be our set of stimulus data. Further define *U*(𝒮) as the universal input feature space for our stimuli 𝒮. We used GloVe word embeddings of our stimulus data for *U*(𝒮). For each representation *t* ∈ 𝒯, we generated an encoder *E*_*t*_(·) such that the encoder extracts only information in *U*(𝒮) that is needed to predict *t*(𝒮). We did this by using a bottlenecked linear neural network that maps every **u** ∈ *U*(𝒮) to an intermediate low-dimensional latent space 𝓛_*t*_ = *E*_*t*_(*U*(𝒮)) and then maps it to the given representation space,

$$\forall s\in 𝒮,t\left(s\right)\approx f\left(E_{t}\left(U\left(s\right)\right)\right)$$

where *f*(·) is mapping from 𝓛_*t*_ to *t*(𝒮).

We used a small latent space of 20 dimensions to encourage the encoder to extract only the information in *U*(𝒮) that is relevant to compute *t*(𝒮). These latent spaces were then scored on how much better they transferred to other representations. The use of this approach over simple linear regression enables us to normalize representations by their dimensionality and measure the overall generality of each representation rather then the total amount of information contained in each representation, which is more dependent on the total number of dimensions in each representation. For every pair of representations (*t*_1_, *t*_2_) ∈ 𝒯, we next generate a decoder *D*_*t*_1_→*t*_2__ such that *D*_*t*_1_→*t*_2__(𝓛_*t*_1__) = *D*_*t*_1_→*t*_2__(*E*_*t*_1__(*U*(𝒮))) approximates *t*_2_(𝒮). This yields a total of *n*^2^ decoders, where *n* = |𝒯| is the total number of representations. All networks were trained with batches of size 1024 and standard stochastic gradient descent with a learning rate of 10^−4^ for the initial encoders and 2 × 10^−5^ for the decoders. We enforce a strict linearity constraint on both the encoder and decoder to ensure that representations that are nonlinearly decodable from one another are treated as distinct (Naselaris et al., 2011). Hyperparameters were chosen via coordinate descent.

We finally used the decoders to generate a pairwise *tournament matrix* **W***_t_* for each representation *t* by “fighting” all pairs of decoders that output to representation *t* using a held-out test set 𝒮_*test*_ of sentences. Element (*i*, *j*) in **W**_*t*_ contains the ratio of samples in the test set for which *D*_*t*_*i*_→*t*_ has lower mean squared error than *D*_*t*_*j*_→*t*_, that is,

$$W_{t_{\left(i,j\right)}}=\frac{{𝔼}_{s\in {𝒮}_{\text{test}}}\left[D_{t_{i}\to t}\left(s\right)<D_{t_{j}\to t}\left(s\right)\right]}{{𝔼}_{s\in {𝒮}_{\text{test}}}\left[D_{t_{i}\to t}\left(s\right)>D_{t_{j}\to t}\left(s\right)\right]}.$$

For example, if the decoder *D*_*A*→*C*_ has lower mean squared error than decoder *D*_*B*→*C*_ for 75% of the data in 𝒮_*test*_, we assign the ratio of 0.75/0.25 = 3 to entry (*A*, *B*) in the tournament matrix **W**_*C*_ for representation *C*.

We then averaged these pairwise tournament matrices **W***_t_* over all *t* to generate an average pairwise tournament matrix W* which encodes the average relative performances of each representation in transferring to the other representations in our set. Further averaging this matrix along its first axis yields a metric of the relative propensity of each representation to transfer to each other representation *in general*. We used this metric to denote the *generality* score of a representation.

Finally, we isolated the pairwise tournament matrix of an intermediate representation from a machine translation model from English to German. We similarly averaged this matrix along its first axis to yield a metric of *translation* transfer performance for each representation that was not from the English to German model.

### Voxelwise Variance Partitioning

For voxelwise variance partitioning, we used the method established by de Heer et al. (2017). When partitioning the variance explained between two input spaces, *A* and *B*, over an output set of voxels, we generated three models per voxel *v* and subject *x*: $\hat{B}$_(*x*,*v*,*t*_*A*_)_, $\hat{B}$_(*x*,*v*,*t*_*B*_)_, and $\hat{B}$_(*x*,*v*,*t*_*A*·*B*_)_. $\hat{B}$_(*x*,*v*,*t*_*A*_)_ and $\hat{B}$_(*x*,*v*,*t*_*B*_)_ refer to the models generated by using only *A* or *B* respectively, as the input representation. $\hat{B}$_(*x*,*v*,*t*_*A*·*B*_)_ refers to the model generated by using *A* concatenated with *B* as the input representation.

Variance explained was computed on a held-out pair of test stories from our podcast data. Variance explained by the concatenated model but not explained by a single model was inferred to be uniquely explained by the other single model. Only language responsive voxels where at least 5% of the response variance for the test story was explainable (Nishimoto et al., 2017) were included in our variance partitioning analyses.

## RESULTS

### Correlations Between Encoding Performance and Other Metrics on Language Representations

Several recent studies (Caucheteux et al., 2021a; Schrimpf et al., 2021) have shown that language models whose representations perform better as encoding model inputs tend to perform better at predicting upcoming words or sentences. We first sought to replicate this result by examining the relationship between encoding performance and the ability of a representation to predict next words. We extracted a total of 97 representations from several different natural language processing (NLP) models, including three word embedding spaces (GloVe, BERT-E, and FLAIR; Akbik et al., 2019; Devlin et al., 2019; Pennington et al., 2014), three unidirectional language models (GPT-2 Small, GPT-2 Medium, and Transformer-XL; Dai et al., 2019; Radford et al., 2019; Wolf et al., 2019), two masked bidirectional language models (BERT and ALBERT; Devlin et al., 2019; Lan et al., 2019), four common interpretable language tagging tasks (named entity recognition, part-of-speech identification, sentence chunking, and frame semantic parsing; Akbik et al., 2019), and two machine translation models (English → Mandarin, English → German; Tiedemann & Thottingal, 2020). A full description of each of these representations is given in the Supporting Information, which is available at https://doi.org/10.1162/nol_a_00087.

**Figure 1.**
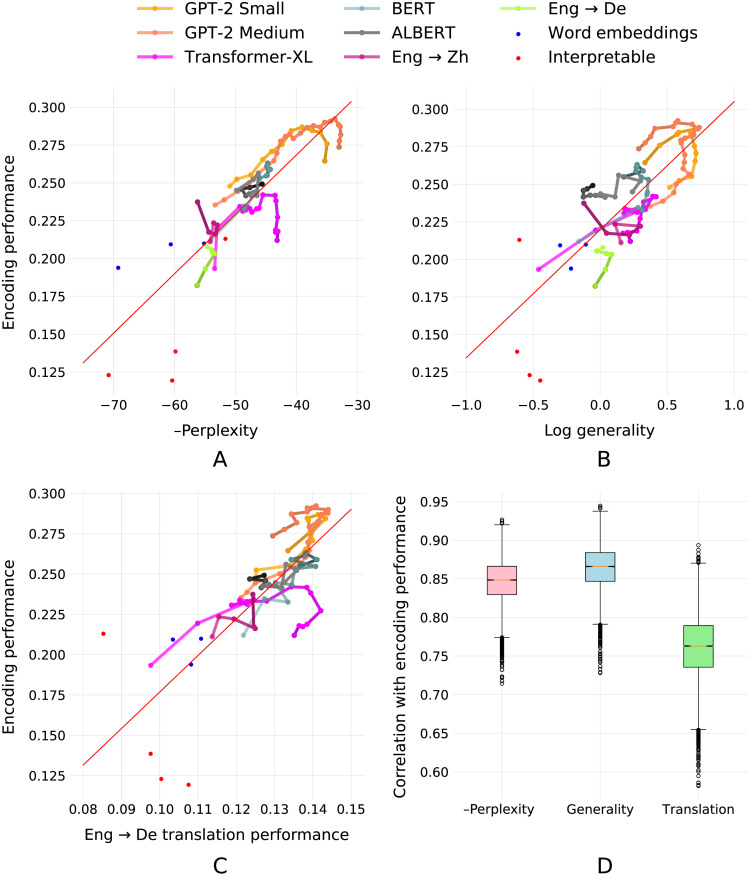
Correlates of encoding performance. Plotted are 97 language representations as measured according to four metrics: (A) Average encoding performance across five subjects, next word prediction performance, shown here as negative perplexity; (B) general transfer performance to other representations; and (C) transfer performance to a representation extracted from an English-to-German translation model. In each plot, encoding performance is compared to one of the other metrics. In every case, encoding performance of a representation correlates strongly with the other metric. Additionally, representations extracted from unidirectional language models (GPT-2 Small and GPT-2 Medium) are the highest in each of these metrics. This suggests that the reason features from unidirectional models such as GPT-2 (shown in orange) perform well may be because they are generally good features that perform well when transferring to other language representations, rather than because they are simply good at next word prediction. (D) Subsamples. To robustly estimate correlations, 70 points from each comparison were selected at random 10,000 times and then correlated. These are presented in the boxplot.

Using a natural language fMRI data set, we constructed voxelwise encoding models for each of the 97 language representations. For each voxel, we then computed the *encoding performance* as the correlation between predicted and actual BOLD responses on a held-out test data set. We measured the overall encoding performance for each representation by computing the average encoding performance across all language-responsive voxels. We then measured how well each representation can do next word prediction by computing a “linearly extractable perplexity” score (see Materials and Methods). Comparing encoding performance and next word prediction performance across the 97 representations showed that these metrics are have high mean correlation (*r* = 0.847; Figure 1A), replicating earlier results (Caucheteux et al., 2021a; Schrimpf et al., 2021).

While the high correlation between next word prediction performance and encoding performance is argued to be evidence for predictive coding in the brain, an alternative hypothesis is that certain representations work well as encoding models because they contain information that is generally useful for predicting representations from many language tasks, including next word prediction. To test this hypothesis, we measured how well each of the 97 representations could predict, or “transfer to” the other 96 representations (see Materials and Methods). This yields a metric measuring *general transfer performance* or *representational generality*. This metric tells us how much generally useful language information is contained in each representation as compared to the other representations. Representations that contain information useful for explaining other representations will have higher generality values, while those that contain little useful information will have lower values. An extended discussion of this metric and the motivation behind it is given in the Supporting Information, which is available at https://doi.org/10.1162/nol_a_00087.

Figure 1B shows that there exists a very strong mean correlation (*r* = 0.864) between how well a representation transfers in general and its encoding performance. This correlation is numerically greater but not significantly different from the correlation between encoding performance and next word prediction performance. This result provides support for the hypothesis that certain representations produce effective encoding models because they have high general transfer performance, but does not constitute proof. Indeed, the high correlation between all three metrics—next word prediction performance, general transfer performance, and encoding performance—makes differentiation between competing causal hypotheses difficult. Yet even this confusion raises a salient point: Correlation between these metrics is not sufficient to support a causal argument.

To further illustrate the difficulty of making causal claims based on this type of evidence, we present a final example of the same type which is absurd on its face. In this third analysis, we compared encoding performance for each representation to one specific type of transfer performance: the ability of each representation to predict features extracted from an English-to-German translation model (Tiedemann & Thottingal, 2020; see Materials and Methods). From the set of models used to compute our representational generality metric, we isolated those that predicted the intermediate representation of a machine translation model that was trained to convert English text to German text. We then computed the relative transfer performance of each of our representations to this machine translation representation, yielding a metric we call “*Eng* → *De translation transfer performance*.” Comparing encoding performance to Eng → De translation transfer performance again showed a high mean correlation (*r* = 0.780; Figure 1C). How should we interpret this result? If we were to assume that this correlation suggests causation (and were not aware of the other results), we might conclude that the objective underlying the brain’s processing of English language is translation to German. But this is absurd, not least because none of the subjects in this study speak fluent German. Instead, we should conclude that this correlation—like the others we have reported here—is likely the result of common causes. To effectively predict brain responses, a representation must contain many different types of linguistic information. Some types of linguistic information are useful for predicting representations extracted from an Eng → De translation model. Thus, representations that make for good encoding models also excel at translating English to German.

### Comparing Across Layers of Neural Network Language Models

We next investigated implications of predictive coding theory just within a single NNLM. One consequence of predictive coding theory is that the brain should encode information about its next word predictions. Thus, representations that contain predictive information about next words should explain brain responses well. Further, representations that can predict next words should uniquely explain some variance in brain responses that is not explained by representations that lack that predictive information. We investigated this issue by analyzing encoding performance for different layers from two variations of the same NNLM, GPT-2 Small and GPT-2 Medium (Radford et al., 2019). In these unidirectional language models, words enter at the first layer and then propagate through many intermediate layers until, at the last layer, the model predicts the next word. Across layers, the representations slowly shift from more input-like in the early layers to more prediction-like in the latest layers. Many earlier reports have shown that the best encoding performance (and transfer performance) is obtained from layers closer to the middle of such a model, and not the latest layers (Antonello et al., 2021; Caucheteux et al., 2021a; Caucheteux & King, 2022; Jain & Huth, 2018; Toneva & Wehbe, 2019). This suggests that the intermediate layers are better at capturing linguistic structure than the latest layers, even though the latest layers are best at next word prediction. This could contradict predictive coding theory, which would suggest that the latest layers, which are best at predicting future words, should also yield the best encoding models.

**Figure 2.**
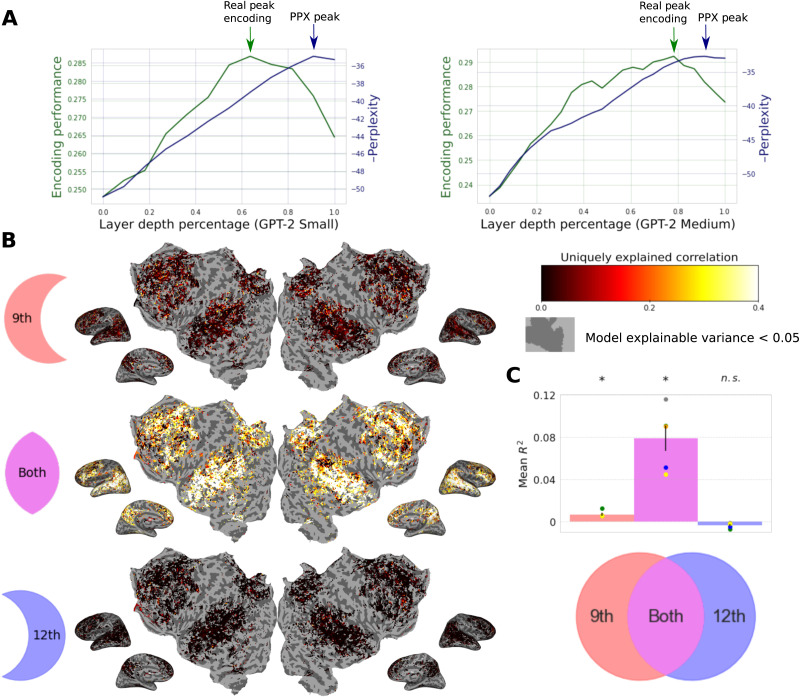
Variance partitioning is performed on the encoding performance of encoding models built from GPT-2. (A) A plot showing the change in encoding performance as a function of layer depth in GPT-2 Small and GPT-2 Medium. (B) Maps showing the individual contribution of variance explained from each component of a joint GPT-2 Small encoding model. (C) A mean breakdown of the contribution of variance of each component of this model. The 12th layer explains no unique variance above the 9th layer despite better next word prediction performance. *R*^2^ is computed as *R* * |*R*| to allow for negative values.

To study this issue more closely, we both constructed encoding models and measured next word prediction performance for each layer of the two GPT models. Figure 2A shows the next word prediction performance of each layer alongside the hypothesized relationship between encoding performance and depth suggested by predictive coding. As expected, the next word prediction performance increases nearly monotonically, achieving its highest values in the latest layers. However, actual encoding model performance (averaged across voxels and subjects) does not follow this pattern. Here, consistent with earlier reports, we see that encoding performance peaks at between 60% and 80% of maximum model depth, and then falls precipitously for the latest layers. If the brain was truly representing predictions for the next word, we should not see this pattern.

However, this overall comparison is not conclusive: Although the intermediate layers provide better average encoding performance, it is still possible that the latest layers, by virtue of doing better next word prediction, uniquely capture some variance in brain responses. This would be sufficient to support the theory of predictive coding, which does not require that every brain area represent next word predictions, only that some do. Put succinctly, next word prediction anywhere supports predictive coding everywhere. To explicitly test for this possibility we used a variance partitioning analysis to determine whether any brain responses are uniquely explained by the last layer. In this analysis, we measured how much of the variance in brain response could be uniquely explained by either the most performant layer in each model (measured by average voxelwise correlation) or the last layer in each model, as well as the amount of variance that could be explained equally well by either of those layers. This was done by fitting three encoding models: one with just the best layer, one with just the last layer, and one with both representations concatenated.

Figure 2B and C show the results of this variance partitioning analysis. Here we see that the most performant layer (the ninth layer in GPT-2 Small) does not merely outperform the last layer, but actually *dominates* the last layer across the entire cortex. While much of the variance that can be explained by either layer is explained by both, the last layer uniquely explains no significant additional variance above the ninth layer, while the ninth layer explains some variance above the last layer. In fact, owing to the combination of high covariance of the 12th layer features with the ninth layer features and having low beneficial contribution of its own, the ridge regression using the concatenated model performs slightly worse than the ridge regression using just the ninth layer features. This leads to a negative average measured unique variance explained for the 12th layer, which can be seen in Figure 2C.

If the brain was performing an internal prediction task, then we would expect that at least some voxels would have unique variance that could be explained only by the last layer, which is most similar to the final predictive output of the language model. The fact that no variance is uniquely explained by the last layer suggests that some intermediate structural representation that is reached in the course of next word prediction is closer to what the brain internally represents. As the intermediate layers are also the best at transferring to other representations, this further supports the hypothesis that overall representational generality—and not next word prediction—underlies the success of language models at predicting brain data.

## DISCUSSION

Recent work has argued in favor of a predictive coding theory of linguistic cognition based on evidence from encoding models (Schrimpf et al., 2021). Among the most noteworthy claims stemming from the encoding model literature is the observation, which we have replicated, that a strong correlation exists between the encoding performance of a linguistic representation and its ability to predict next words. This correlation has been taken as causal evidence that the brain is driven by predictive mechanisms that underlie its high-level objectives. We believe, however, that this inference is flawed. It is perfectly reasonable to expect that *if the brain encodes a feature, then a model that also encodes the same feature will fit the brain better than a model that does not, all other things equal*. But predictive coding arguments apply this implication in the wrong direction by assuming that *models that fit the brain better than others have feature X, so therefore the brain also has feature X*, where “X” in this case is next word prediction. Issues with this particular type of reasoning about artificial and biological computation are discussed extensively by Guest and Martin (2021).

As an analogy, consider the problem in signal processing of spectral density estimation. Linear autoregressive models are often used to provide regularized estimates of the spectrum of a signal (Ulrych & Bishop, 1975). Yet it would be false to suggest that spectral density estimation is an example of predictive coding, as autoregressive models are merely one way to accomplish this goal. In the same way, we cannot assume that language models fit the brain well because the brain is trying to predict future inputs. The correlation between a representation’s encoding performance and its ability to transfer to an English-to-German translation representation underscores this problem. If we were to apply the same logic to this correlation as is applied to the correlation between the predictive power of models and their encoding model performance, we might—absurdly—conclude that what underlies linguistic processing in the brain is German translation. Yet a much simpler explanation for both effects is that generality in transferring to linguistic tasks is highly correlated with both measures, and representations that are suitable for one sufficiently general task (such as language modeling) are likely to be suitable for many others (such as translation or brain encoding).

Furthermore, one possible entailment of predictive coding theory is that representations that better encode next word prediction ought to capture some responses somewhere in the brain better than representations that do not. However, our variance partitioning analysis showed that as next-word linear decodability continues to improve across layers in GPT-2 Small, encoding performance declines not merely on average, but everywhere.

One might object to an argument such as this, on the basis that such an entailment is not necessary for predictive coding and that prediction may simply be an objective of the language system, or that prediction in the brain occurs not at the word level but at a more abstract conceptual level. While this seems exceedingly plausible, we are somewhat wary of treating predictive coding itself as a rigorous scientific theory if it is only to mean that the brain uses the objective of (possibly conceptual) prediction in order to help generate or manifest the language system. We feel that this interpretation of predictive coding is vague and underdefined, as it is unclear to us what provably false statements about the nature of the language system could be made if directly measurable quantities such as linear next-word prediction performance are rejected as irrelevant. We acknowledge that the tests we have explored here may not be suitable for assessing every potential interpretation of predictive coding. Thus, we would encourage our peers in the field who hold affirmative views regarding “conceptual” predictive coding to expand and formalize them, so that they can be more precisely evaluated.

Of course, it is possible that the effects of predictive coding are simply undetectable at the spatial and temporal resolution of fMRI, and that is a fundamental limitation of the analyses in this article. But suppose that we could do this variance partitioning analysis at perfect resolution, without the limitations of neuroimaging methods, limited data, and imperfect regression techniques. If we still observed no meaningful improvement anywhere in the brain from adding a later layer of a language model to an earlier one, then proponents of predictive coding would surely need to specify what quantifiable and falsifiable claims are being made about the language system according to predictive coding theory that uniquely distinguish prediction from absurd objectives like English-to-German translation.

Encoding model arguments concluding that the brain learns through prediction must necessarily contend with the possibility that observed phenomena are the product of the low-dimensional structure that naturally arises across language representations (Antonello et al., 2021), whether they be from the brain or artificial models, and not the consequence of an inherently predictive process. Furthermore, eliminating the confounds between structure and prediction is extremely challenging, as any sufficiently structured linguistic system will necessarily contain some predictive information, and any sufficiently predictive linguistic system will possess inherent structure.

What does this all mean for the wider claims about a theory of predictive coding for linguistic processing? We do not believe any of the results or arguments made in this article should be considered evidence *against* predictive coding as a cognitive theory. Indeed, predictive coding elegantly and mechanistically explains many observed phenomena. We do, however, claim that evidence from encoding model research should not be seen to currently *support* a theory of predictive coding. This is due to the fact that much of what is cited as the strongest evidence in favor of predictive coding from encoding model research would very likely be true even in the absence of predictive coding, as our representational generality results demonstrate.

If we are to reject the existing evidence, a logical next question is What would suffice as evidence for predictive coding? One possible avenue might be to determine whether next word information can be used to predict brain activity before word onset better than information from previous words. This is exactly the approach taken by Goldstein et al. (2021) and Caucheteux et al. (2021b). They showed that a small but statistically significant improvement in encoding performance can be gleaned by using future words to predict brain responses, as compared to only using past words. While this is an elegant test, we feel the conclusion that is drawn—that this implies that predictive coding occurs in the brain—should still be viewed with skepticism. This is because it is challenging to differentiate between next word predictive information that is *incidentally* useful for prediction but was generated for some other objective, and information that has been gleaned in the process of *directly* trying to predict next words. As we have seen, linguistic information is highly versatile and general, and information that is useful for one task is often useful for many others. Recall, for instance, that it is entirely possible to build a reasonably effective encoding model for English speakers using information derived from an English-to-German translation model. So it is quite reasonable to believe that some predictive or future information would be useful for brain encoding even if prediction itself is not the driving mechanism of linguistic processing in the brain.

If evidence suggesting that next word information aids in brain encoding does not suffice, what might? Predictive coding as a theory seems, ironically, to not predict many phenomena uniquely. Much of what predictive coding can explain can also be explained without it. So what measurable phenomenon differentiates a world where the brain does predictive coding from one where the brain does not? The discovery of some naturally occurring low-level neural circuit that encodes prediction as an objective of language learning would be strong evidence. There is undeniably much existing evidence that is necessary for predictive coding to be true. But without direct access to the neural circuits underlying language processing, convincingly sufficient evidence for predictive coding will no doubt be difficult to produce. Cognitive theories invoking prediction as an essential element are fundamentally tied to those that invoke generality, or more simply, learned structure, as each can plausibly explain the other. There may be no easy path forward in disentangling these concepts.

Predictive coding presents both a promise and a challenge to computational neurolinguists. On one hand, as a cognitive theory, it makes a relatively concrete and exceedingly plausible claim about the high-level nature of the brain that greatly coincides with our intuition. It would plainly represent a grand achievement of modern computational neuroscience if it could be proven to be true. On the other hand, serious inquiry into predictive coding naturally introduces a perfidious tangle of confounds. Finding a solution to these confounding issues may be a major step toward discovering the computational principles underlying language processing in the human brain.

## ACKNOWLEDGMENTS

We would like to acknowledge Shailee Jain and Arjun Bose for editing and feedback on this manuscript. This research was funded by grants from the NIDCD and NSF (1R01DC020088-001), the Burroughs-Wellcome Foundation, and a gift from Intel Inc.

## FUNDING INFORMATION

Alexander Huth, Burroughs Wellcome Fund (https://dx.doi.org/10.13039/100000861). Alexander Huth, Intel Corporation (https://dx.doi.org/10.13039/100002418). Alexander Huth, National Institute on Deafness and Other Communication Disorders (https://dx.doi.org/10.13039/100000055), Award ID: 1R01DC020088-001.

## AUTHOR CONTRIBUTIONS

**Richard Antonello**: Conceptualization: Lead; Funding acquisition: Supporting; Investigation: Lead; Methodology: Lead; Software: Lead; Validation: Lead; Visualization: Lead; Writing—original draft: Lead; Writing—review & editing: Equal. **Alexander Huth**: Conceptualization: Supporting; Data curation: Lead; Formal analysis: Lead; Funding acquisition: Lead; Investigation: Supporting; Methodology: Supporting; Project administration: Lead; Resources: Lead; Software: Supporting; Supervision: Lead; Validation: Supporting; Visualization: Supporting; Writing—original draft: Supporting; Writing—review & editing: Equal.

## Supplementary Material



## TECHNICAL TERMS

Predictive coding: A neuroscientific theory that posits that the brain uses a prediction objective or error to efficiently learn.
Language models: Autoregressive machine learning models that are trained to predict next words given a previous context.
Perplexity: A formal metric for how well a language model can predict a given data set; lower is better.
Encoding models: Machine learning models that predict brain response from natural stimulus features.
