# Supplementary material for: Predictive Coding or Just Feature Discovery? An Alternative Account of Why Language Models Fit Brain Data
Source: Neurobiol Lang (Camb). 2024 Apr 1;5(1):64–79. doi: 10.1162/nol_a_00087 (PMC11025645; doi:10.1162/nol_a_00087)
Supplement: Supplementary file 1 [file nol-5-1-64-s001.pdf]

Supplementary Material

Representation Descriptions

Descriptions for each of the representations used in our encoding model performance analysis (Figure 1) are given below:

GloVE (Pennington et al., 2014) is a 300-dimensional word embedding space. It is an dimensionality-representation representation of word-word co-occurrence statistics. GloVe embeddings were sourced from `http://nlp.stanford.edu/data/glove.6B.zip`

BERT-E (Devlin et al., 2019) is a 3072-dimensional contextualized word embedding space extracted from BERT. We used the Flair NLP (Akbik et al., 2019) implementation of BERT embeddings.

FLAIR (Akbik et al., 2019) is a 4096-dimensional contextualized character level word embedding space. We used the Flair NLP implementation of this word embedding space.

POS is the 53-dimensional representation stored at the pre-softmaxed output logit layer of the pre-trained FLAIR (Akbik et al., 2019) part-of-speech LSTM-based sentence tagger.

CHUNK is the 45-dimensional representation stored at the pre-softmaxed output logit layer of the pre-trained FLAIR (Akbik et al., 2019) LSTM-based sentence chunker.

NER is the 76-dimensional representation stored at the pre-softmaxed output logit layer of the pre-trained FLAIR (Akbik et al., 2019) LSTM-based named entity recognition sentence tagger.

FRAME is the 5196-dimensional representation stored at the pre-softmaxed output logit layer of the pre-trained FLAIR (Akbik et al., 2019) LSTM-based semantic framing (verb disambiguation) tagger.

The FlairNLP repository which we used for the preceding representations can be found here: `https://github.com/flairNLP/`

GPT-2 Small is a set of 12 representations, each 768-dimensional, built from each hidden state output from the 12 transformers that compose the GPT-2 Small unidirectional

language model (Radford et al., 2019). Representations were built using a sliding window of 64 words as a context. We used the HuggingFace (Wolf et al., 2019) implementation of this network to extract feature for these representations.

GPT-2 Medium is a set of 24 representations, each 1024-dimensional, built from each hidden state output from the 24 transformers that compose the GPT-2 Medium unidirectional language model (Radford et al., 2019). Representations were built using a sliding window of 64 words as a context. We used the HuggingFace (Wolf et al., 2019) implementation of this network to extract feature for these representations.

Transformer-XL is a set of 18 representations, each 1024-dimensional, built from each hidden state output from the 18 transformers that compose the Transformer-XL unidirectional language model (Dai et al., 2019). Representations were built using a sliding window of 64 words as a context. We used the HuggingFace (Wolf et al., 2019) implementation of this network to extract feature for these representations.

BERT is a set of 12 representations, each 768-dimensional, built from each hidden state output from the 12 transformers that compose the BERT bidirectional masked language model (Devlin et al., 2019). Representations were built using a sliding window of 64 words as a context, with the last non-special token being the designated mask token. We used the HuggingFace (Wolf et al., 2019) implementation of this network to extract feature for these representations.

ALBERT is a set of 12 representations, each 768-dimensional, built from each hidden state output from the 12 transformers that compose the ALBERT bidirectional masked language model (Lan et al., 2019). Representations were built using a sliding window of 64 words as a context, with the last non-special token being the designated mask token. We used the HuggingFace (Wolf et al., 2019) implementation of this network to extract feature for these representations.

Eng ⇒ Zh is the set of 6 encoder hidden state representations, each 512-dimensional, of the HuggingFace/Helsinki-NLP (Tiedemann and Thottingal, 2020; Wolf et al., 2019)

implementation of a Transformer-based machine translation model from English to Mandarin Chinese. Representations were built using a sliding window of 64 words as a context. The pretrained network we used can be found here: `https://huggingface.co/Helsinki-NLP/opus-mt-en-zh`.

Eng $\Rightarrow$ **De** is the set of 6 encoder hidden state representations, each 512-dimensional, of the HuggingFace/Helsinki-NLP (Tiedemann and Thottingal, 2020; Wolf et al., 2019) implementation of a Transformer-based machine translation model from English to German. Representations were built using a sliding window of 64 words as a context. The pretrained network we used can be found here: `https://huggingface.co/Helsinki-NLP/opus-mt-en-de`.

Encoding Performance Reference

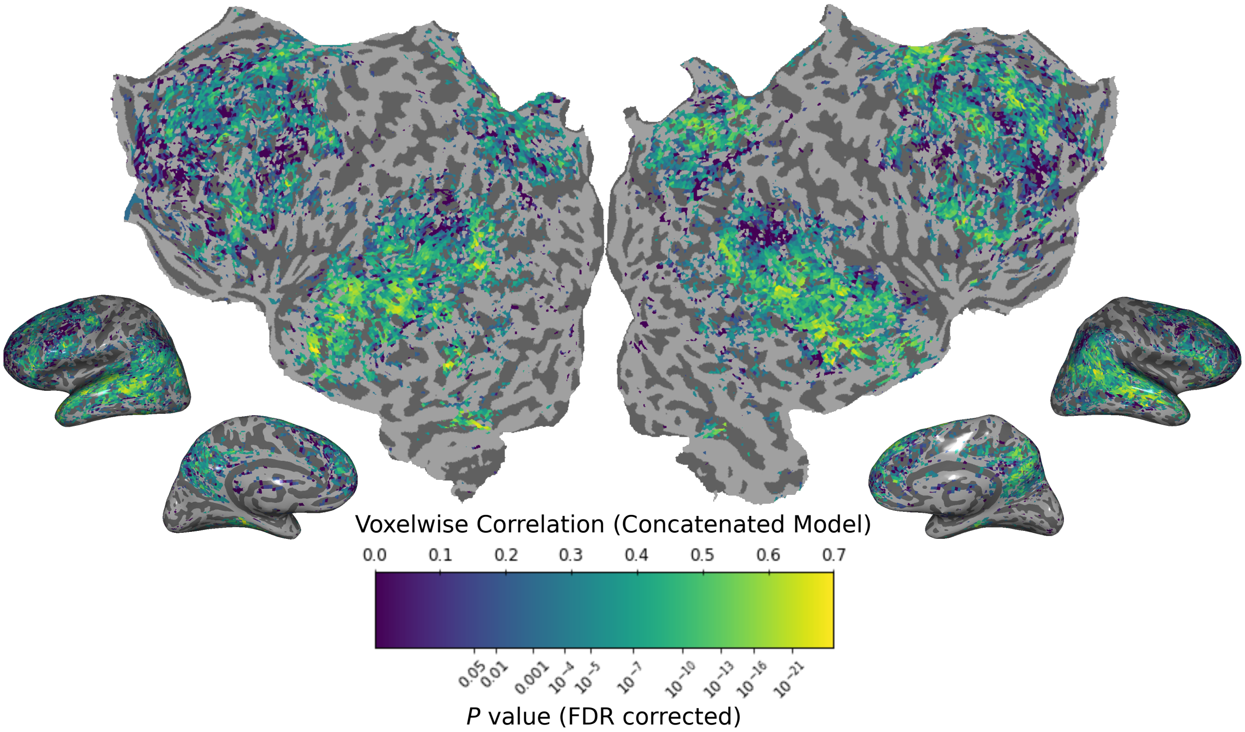

Figure S1

Shown above is a reference voxelwise map of encoding performance with p-values of a model trained jointly on the concatenated representations from the 9th and 12th, the model that is referred to as "**Both**" in Figure 2. P-values were computed using a false-discovery-rate-corrected permutation test on the test responses. Small p-values were extrapolated using a parametric bootstrap with a Fischer transform on the correlations. Only voxels which have an explainable variance (Nishimoto et al., 2017) of at least 0.05 are included.

Motivating Generality

In this supplemental discussion, we give an extended motivation behind the *generality* metric from the main work, and explain why it is a natural candidate as a correlate for encoding model performance. We also briefly justify why we chose this metric over direct task performance, as others have done (Schrimpf et al., 2021).

To understand why the proposed generality metric is a natural predictor of encoding performance, we begin by formalizing some of the terms that we have only used informally to this point. Define F as the set of all functions that take as input a string of words and have a rational output. Further, define R_n as the set of all subsets of F that have cardinality n. Note that the set of encoded language representations from our paper is simply a subset of R_{20}. F is infinite and naturally contains both functions that are behaviorally relevant for humans interacting with language ("Does the input string contains a place name?") and functions that are probably not ("What is the number of vowels in the input string, modulo 13?"). Since behavioral relevance is determined by complex social, cultural, and biological factors and will differ between individuals, let us set aside whether we can quantify it and settle for the assumption that such a quantification could exist: for each function over language $f \in F$, there is a corresponding value of "behavioral relevance" associated with that function, $\rho(f)$. Let us also assume that there is an total ordering over this relevance metric, and that for all pairs of functions $f_1, f_2 \in F$, there is a truth value for $\rho(f_1) > \rho(f_2)$; in other words, some functions are more "relevant" than others. Similarly, let us assume that we can extend this notion of behavioral relevance to members of R_n as well. As a relevant aside, this high-level notion of "behavioral relevance" bears similarity to modern "teleosemantic theories" of mental content (Schulte and Neander, 2022).

Let us now compare this to how our "generality" metric works. For any two representations $r_i, r_j \in R_n$, we can compute a prediction performance score $S_j(r_i)$ of how well the outputs of functions in r_i can be used to predict the outputs of functions in r_j. Since some functions are idiosyncratic ("Does the string contain a specific word X?") and

others are trivial (constant functions), we can normalize these scores by computing how much better, relatively, some representations are at predicting other representations: given $r_1, r_2, r_3 \in R_n$, is $S_3(r_1) > S_3(r_2)$? If we consider all of R_n, including both behaviorally relevant and irrelevant functions, there ought to be no representations that are overall better at predicting all other representations. That is, there is no representation $r_{max} \in R_n$ for which $S_{avg}(r_{max})$ is maximized, where S_{avg} is the average prediction performance score over all members of R_n. However, we need not consider the average score of all the members of R_n, but only the members of R_n that have a high behavioral relevance.

We cannot mathematically define behavioral relevance, but we can still approach this problem by sampling behaviorally relevant functions that have been defined in other works. In an earlier work (Antonello et al., 2021), we analyzed a sample of behaviorally relevant representations, and found that the organization of these representations (i.e., which representations can predict each other well and which cannot) is recapitulated in the brain. Furthermore, they found that the space of representations has low-dimensional structure. This suggests that the set of linearly independent functions in F that are highly behaviorally relevant is finite and small.

Tautologically, in order for a human to successfully interact using language, the set of functions that their brain computes—a subset of F—must contain at least some functions that have high relative "behavioral relevance". It is not logically necessary for all of the functions that brains computes to have high behavioral relevance. But if this is the case, then the brains is wasting its finite space and energy on neural circuits that have little value. We might reasonably assume that there are evolutionary pressures on brains to minimize this, and thus it is also reasonable to assume that most language-responsive neural circuits compute functions that have high behavioral relevance. So encoding models built from representations that better encode this same set of functions are liable to be better at predicting brain response, as they share more in common with the functions computed in the brain. From this we can suggest that representations that compute many independent

functions with high average behavioral relevance are likely to be good at encoding brain responses.

So now, let us consider a score function $S_{bavg}(r_i)$ which is the average over all prediction performance scores for r_i over the members of R_n that have high behavioral relevance. This score will be high for representations that are useful for predicting many other behaviorally relevant representations, and low for representations that are idiosyncratic or uninformative. Further, a high value for $S_{bavg}(r_i)$ should also entail that r_i is a good encoding model for brain data, since the functions computed by the brain are likely to be in the set that S_{bavg} is averaging over. The "generality" score that we introduce in this paper is simply a finite approximation to S_{bavg}, which is formed by sampling from the set of behaviorally relevant representations, computing S for each pair, and then computing the average score for each representation. This approximation will be close to the actual measure if the sampled representations are representative and sufficiently span the full set of behaviorally relevant functions. Thus, if the assumptions of this framework are met, it is natural to hypothesize that "generality" should closely mirror encoding performance.

Comparing Other Metrics to Generality

How does Next Word Prediction Relate to Generality?

So how does next word prediction fit into this framework? If we are to claim that it is possible for predictive coding to not be correct, we must still explain why representations that are better for next word prediction typically have higher encoding model performance than other representations. Let us define a function $NBP(x)$ which takes as input a string x and outputs the rational probability that the *next bit* of information following x is 1. Note that NBP is a function over strings with a rational output, and so $NBP \in F$. Furthermore, note that NBP is simply a idealized generalization of a language model that acts at the finer granularity of bits instead of tokens. We would like to demonstrate that NBP is a special member of F, in that it is *at least as hard to compute* as any other member of F.

Borrowing terminology from computational complexity theory, we might say that NBP is F-complete. This is because any function call $f(x)$ where $f \in F$ can simply be evaluated using a number of calls to NBP equal to the number of bits in $f(x)$, n. Constructively, $f(x)$ can be evaluated as n successive calls of

$$NBP(\texttt{The k'th bit of the function f evaluated on string s is })$$

where the definition of f and value of s are encoded as strings, and k ranges from 1 to n. The value of n, which is unknown for arbitrary $f(x)$, can be found via binary search with $\mathcal{O}(\log n)$ additional calls to NBP.

Now, since NBP is F-complete, it follows that any sufficiently effective approximator of NBP will have to be able to compute at least some functions from F. We can regard a modern-day language model such as GPT-2 such an approximator. While GPT-2 is by no means a perfect approximator of NBP, it is likely that it excels in particular at computing functions from F that we might intuitively regard as "behaviorally relevant". This is a result of being trained on entirely human-generated linguistic data, whose properties and regularities are governed by human behavior. Being able to compute many behaviorally relevant functions from F means that representations from language models are liable to have good encoding model performance, as we have reasoned above.

The observed correlation between next word prediction performance and encoding performance can be explained by the nature of approximators of NBP. As the approximation improves, so too does the ability of the approximator to compute behaviorally relevant functions in F. The excellent encoding performance of representations extracted from the best language models can be viewed as a phenomenon that shares a *common cause* with their next word prediction performance. Under this theory, rather than next word prediction performance causing high encoding performance, both performance values are caused by the propensity of their internal representations to capture a wide variety of behaviorally relevant functions. A similar argument utilizing the hardness of good translation can be made that explains why English to German transfer performance is also a

good encoding performance predictor.

How does Task Performance Relate to Generality?

One might question in our analyses why we chose to focus on our metric of generality and not a more straight-forward metric like directly interpretable task transfer performance. This was an approach taken by Schrimpf et al., 2021 on a set of tasks from the GLUE dataset (Wang et al., 2018). They found that direct task performance on many of these tasks did not significantly correlate with brain encoding performance. At a high level, we might ask why this does not work if generality does - both measures, after all, compute the ability of a representation to encode information about behaviorally-relevant functions from F. However, in the task performance metric, what is being measured is not S_{bavg}. but rather S_t, where t is the decision function associated with the task in question. Why is this difference significant? While generality attempts to approximate an *average* score for the ability of representations to encode behaviorally-relevant functions in F, task performance only encodes the ability of representations to encode *one of many* behaviorally-relevant functions in F. And unlike prediction, none of the GLUE tasks possess the same special hardness properties in the space of F as NBP. Therefore, S_t is not necessarily need a good predictor of S_{bavg}, unlike NBP. By seeking to approximate an *average* score of many functions rather than the value of just a *single*, non-representative one, generality serves as a much better estimator of encoding performance overall.
